# MLC1 protein: a likely link between leukodystrophies and brain channelopathies

**DOI:** 10.3389/fncel.2015.00106

**Published:** 2015-04-01

**Authors:** Maria S. Brignone, Angela Lanciotti, Serena Camerini, Chiara De Nuccio, Tamara C. Petrucci, Sergio Visentin, Elena Ambrosini

**Affiliations:** Department of Cell Biology and Neuroscience, Istituto Superiore di SanitàRome, Italy

**Keywords:** astrocytes, brain edema, calcium, potassium, chloride ions, myelin vacuolation

## Abstract

Megalencephalic leukoencephalopathy with subcortical cysts (MLCs) disease is a rare inherited, autosomal recessive form of childhood-onset spongiform leukodystrophy characterized by macrocephaly, deterioration of motor functions, epileptic seizures and mental decline. Brain edema, subcortical fluid cysts, myelin and astrocyte vacuolation are the histopathological hallmarks of MLC. Mutations in either the *MLC1* gene (>75% of patients) or the *GlialCAM* gene (<20% of patients) are responsible for the disease. Recently, the GlialCAM adhesion protein was found essential for the membrane expression and function of the chloride channel ClC-2 indicating MLC disease caused by mutation in *GlialCAM* as the first channelopathy among leukodystrophies. On the contrary, the function of MLC1 protein, which binds GlialCAM, its functional relationship with ClC-2 and the molecular mechanisms underlying MLC1 mutation-induced functional defects are not fully understood yet. The human *MLC1* gene encodes a 377-amino acid membrane protein with eight predicted transmembrane domains which shows very low homology with voltage-dependent potassium (K^+^) channel subunits. The high expression of MLC1 in brain astrocytes contacting blood vessels and meninges and brain alterations observed in MLC patients have led to hypothesize a role for MLC1 in the regulation of ion and water homeostasis. Recent studies have shown that MLC1 establishes structural and/or functional interactions with several ion/water channels and transporters and ion channel accessory proteins, and that these interactions are affected by MLC1 mutations causing MLC. Here, we review data on MLC1 functional properties obtained in *in vitro* and *in vivo* models and discuss evidence linking the effects of MLC1 mutations to brain channelopathies.

## Introduction

Channelopathies are a heterogeneous group of disorders of genetic or acquired origin caused by altered function of ion channel subunits or the proteins that regulate them. These include diseases of the cardiovascular, muscular, respiratory, urinary, endocrine, and immune systems. Due to the fundamental role played by ion channels in neuronal activity, channelopathies are involved in a growing number of nervous system disorders (Kim, 2014 and references therein). Channelopathies leading to dysfunction of astrocytes, the glial cells responsible for the control of ion homeostasis in the central nervous system (CNS), have also been described (Wingerchuk et al., 2007; Vaknin-Dembinsky et al., 2014). The rare leukodystrophy megalencephalic leukoencephalopathy with subcortical cysts (MLC) has recently emerged as a potential brain channelopathy associated to astrocyte dysfunction (Van der Knaap et al., 2012; Lanciotti et al., 2013).

Leukodystrophies comprise a large number of rare genetic disorders that affect mainly the CNS with different etiology and a common target. The European Leukodystrophies Association (ELA) offers a classification including the following types: peroxisomal, lysosomal, vacuolating, hypomyelinating, atypical, undetermined. Against such a heterogeneous etiopathogenesis, the common target is myelin: “either the myelin does not form, degrades, or is too abundant” (from the official ELA site: http://ela-asso.com).

Leukodystrophies have an incidence of 1 in 7,663 live births (Bonkowsky et al., 2010) and manifest themselves mostly during childhood or adolescence. MLC disease, first described in 1995, belongs to the childhood-onset vacuolating leukodystrophy group (Van der Knaap et al., 1995a,b, 1996; Singhal et al., 1996). The incidence of MLC is unknown, although it seems to be more frequent in countries surrounding the Mediterranean basin and in the Indian Agarwal community (Topcu et al., 1998; Ben-Zeev et al., 2002; Singhal et al., 2003). Clinically, MLC is characterized by macrocephaly, deterioration of motor functions with ataxia and spasticity, epileptic seizures and mental decline; minor head trauma or common infections can lead to worsening of clinical conditions often with seizures, prolonged unconsciousness and motor deterioration (Van der Knaap et al., 1995a,b, 1996; Singhal et al., 1996; Topcu et al., 1998; Bugiani et al., 2003). Brain magnetic resonance imaging (MRI) shows diffuse white matter swelling, subcortical cysts (mainly in the temporal and fronto-parietal regions) and, in some patients, severe neurodegeneration (Mejaski-Bosnjak et al., 1997). Analysis of brain biopsies has revealed the presence of liquid vacuoles between the outer lamellae of the myelin sheaths, suggesting defects in lamellae compaction or their splitting along the intraperiod line (Van der Knaap et al., 1996; Pascual-Castroviejo et al., 2005). Reactive and stressed astrocytes with vacuoles and swelling in the end-feet contacting blood vessels have also been observed (Van der Knaap et al., 1996; López-Hernández et al., 2011b). Despite extensive white matter damage, the disease phenotype is less severe than in other leukodystrophies in which similar myelin vacuolation is described (Van der Knaap et al., 2012; Lanciotti et al., 2013).

The first gene responsible for MLC was mapped to chromosome 22qtel and identified as *MLC1* (Topçu et al., 2000; Leegwater et al., 2001). The *MLC1* gene consists of 12 exons with an untranslated first exon, spanning at least 24 kilobases (Steinke et al., 2003). A broad spectrum of MLC1 pathogenic mutations (>60, including missense, splice site, insertions and deletions) have been identified in about 80% of affected individuals (Boor et al., 2006). The mutations are distributed along the whole MLC1 protein sequence and show no clear correlation with the severity of the disease phenotype (Patrono et al., 2003; Montagna et al., 2006). The *MLC1* gene encodes a protein that is mainly expressed in brain astrocytes, particularly at the astrocyte end-feet contacting the blood–brain barrier and the pial membrane. Recently, mutations in a second gene encoding the cell adhesion protein GlialCAM have been found in about 50% of MLC patients not carrying mutations in *MLC1* (<20% of the MLC-affected population; López-Hernández et al., 2011a). GlialCAM is highly expressed in the liver and in the CNS, particularly in neurons and glial cells, where it binds MLC1 (López-Hernández et al., 2011b). The identification of GlialCAM as the molecular chaperon and functional modulator of the chloride channel ClC-2 (Jeworutzki et al., 2014) has allowed for the first time to explain the similarities between brain alterations found in ClC-2 KO mice (Blanz et al., 2007) and those characteristic of MLC patients. Notably, this finding also led to identify MLC disease caused by mutations in the *GlialCAM* gene as the first leukodystrophy among brain channelopathies. Although GlialCAM has been initially identified as the molecular chaperon transporting both MLC1 and ClC-2 to the astrocyte plasma membrane, the relationship between MLC1 and the ClC-2 channel is not fully understood yet. MLC1 protein structural features, molecular interactors (see below) and brain alterations observed in MLC patients (edema, fluid cysts, astrocyte, and myelin vacuolation) suggest that MLC1 can play a role in the regulation of ion and water fluxes and cell volume. However, although several recent studies support this hypothesis (Ridder et al., 2011; Lanciotti et al., 2012; Brignone et al., 2014; Hoegg-Beiler et al., 2014; Dubey et al., 2015), the exact function of MLC1 is still unknown.

In this article we shall present and discuss data on MLC1 structural and functional properties obtained in different experimental settings, suggesting a link between MLC1 mutation-induced alterations in MLC disease and brain channelopathies.

## The Puzzling MLC1 Protein: Biochemical Features and Cellular Localization

### Molecular Structure and Post-Translational Modifications

**FIGURE 1:**
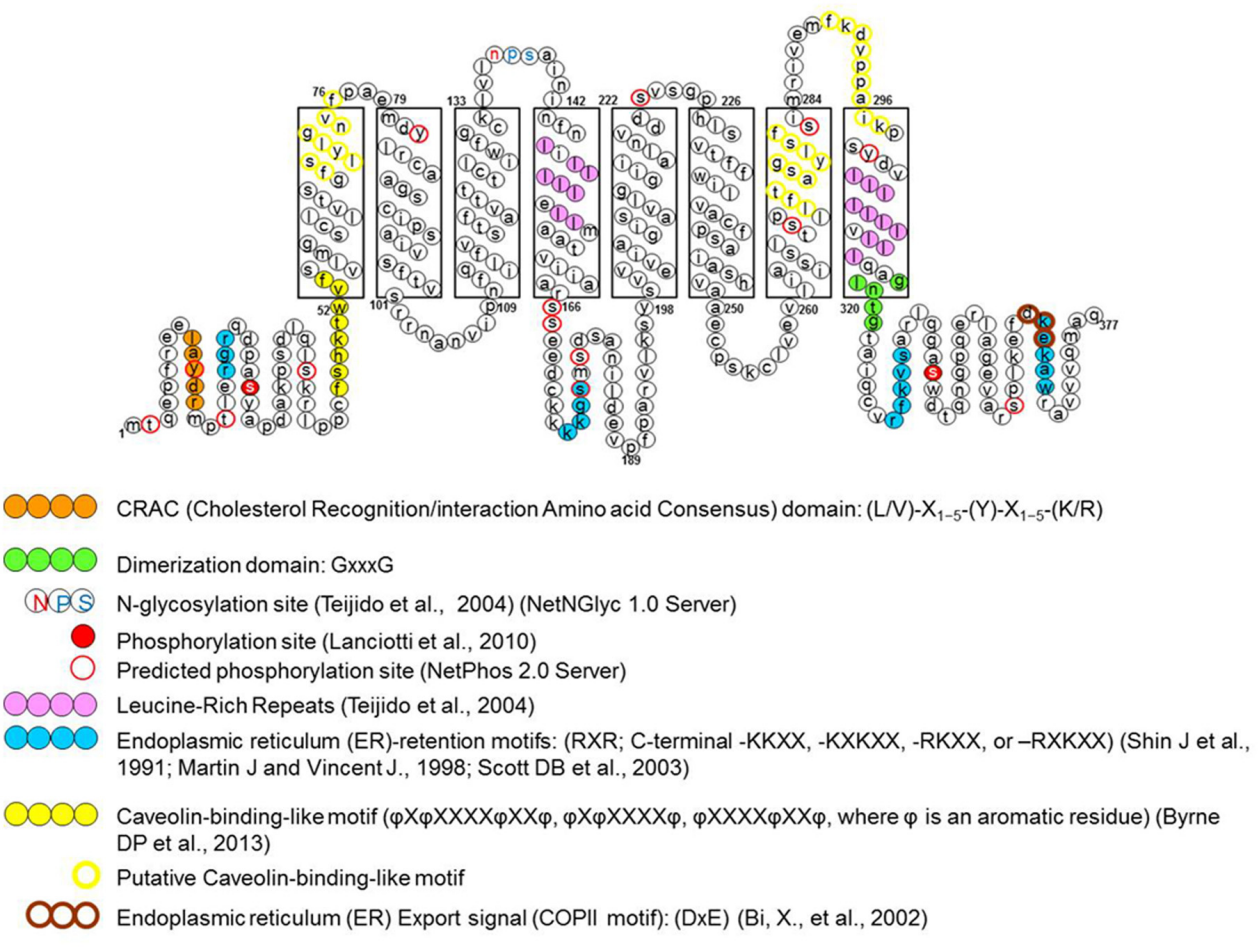
**Schematic representation of specific amino acid consensus motifs found in the human megalencephalic leukoencephalopathy with subcortical cysts (MLC1) protein sequence**. The picture shows consensus motifs for post-translational modifications (glycosylation and phosphorylation), protein–protein or protein–lipid interactions (protein dimerization and cav-1 and cholesterol binding sites) and protein trafficking signals (endoplasmic reticulum export and retention signals), either putative (empty circles), or functionally identified (filled circles) (modified from Lanciotti et al., 2012).

After the discovery of mutations in the *MLC1* gene as the main cause of MLC, several research groups have investigated the physiological function of MLC1 with the aim of disclosing the pathogenic mechanisms underlying the disease. This has been a particularly challenging task. Analysis of the primary nucleotide sequence indicated that the human *MLC1* gene encodes a 377-amino acid highly hydrophobic protein containing eight predicted transmembrane domains and short amino and carboxylic- cytoplasmic tails (**Figure 1**; Meyer et al., 2001; Teijido et al., 2004). MLC1 also contains an internal repeat structure resulting in a partial homology of the primary sequence between the two halves of the protein that is also found in several ion channels (Teijido et al., 2004). However, blast sequence analysis indicates that MLC1 has no similarities with known proteins, with the exception of a very low homology with the shaker-related voltage gated potassium (K^+^) channel Kv1.1 α subunit (less than 20% amino acid identity; Teijido et al., 2004).

Based on this knowledge, the first studies aimed at identifying the biochemical and structural features of MLC1 protein. Early experiments carried out in transiently transfected heterologous cells (HeLa cells and Xenopus oocytes; Teijido et al., 2004), cultured primary rat astrocytes and rat brain tissue (Teijido et al., 2004; Ambrosini et al., 2008) showed that MLC1 is a 36–40 kDa protein that is able to form highly stable dimeric and oligomeric structures (mainly dimers but also tetramers). By performing subcellular fractionations of rat astrocytes and brain tissue it was found that the MLC1 monomeric form is present only in the cytosolic fraction (mainly organelle fraction) while the dimers are associated with the membrane compartments (plasma membrane and endoplasmic reticulum membranes; Ambrosini et al., 2008; Lanciotti et al., 2010). Database analysis of the MLC1 primary sequence revealed potential post-translational modification sites, including phosphorylation and glycosylation sites (**Figure 1**). However, the results of endoglycosydase treatment and site-directed mutagenesis of the putative glycosylation site (NPS in the second extracellular loop, **Figure 1**) ruled out that MLC1 is glycosylated when expressed in heterologous cells (Teijido et al., 2004), but did not allow to exclude that the endogenous protein is glycosylated in astrocytes. *In vitro* studies on recombinant peptides and endogenous protein from rat astrocytes indicated that MLC1 is phosphorylated at its NH_2_ and COOH terminals by both PKA and PKC and PKC alone, respectively (Lanciotti et al., 2010; **Figure 1**). Furthermore, it was found that treatment with substances activating PKC or PKA and phosphatase inhibitors (forskolin, okadaic acid, phorbol esters, genistein) can modify MLC1 plasma membrane expression and formation of multimeric structures (Lanciotti et al., 2010). Interestingly, analysis of the MLC1 aminoacidic sequence revealed the presence of an arginine-based ER retention motif RXR (Schutze et al., 1994; Michelsen et al., 2005) localized in the NH_2_ terminal near the PKC and PKA phosphorylation sites (**Figure 1**). Changes in the extent of phosphorylation at sites adjacent to the RXR-type ER retention motif may enable a given protein to interact with the forward secretory machinery by hiding or hampering the retention signals, therefore allowing channel/receptor trafficking to the plasma membrane (Scott et al., 2003). The ability of intracellular signaling to modulate ER export of RXR-containing protein defines a regulatory pathway that may provide dynamic control over the assembly and surface expression of a wide range of ion channels and receptors (Shikano and Li, 2003). Our data indicating that PKC and PKA activation favors MLC1 expression in the plasma membrane and the presence of an ER retention motif near the PKC/PKA phosphorylation sites suggest this possibility also for the MLC1 protein. The regulation of MLC1 membrane localization (and also activity) by PKA and PKC phosphorylation has been demonstrated for several ion channels and transporters. For example, PKA activation enhances the forward trafficking of the cystic fibrosis transmembrane conductance regulator (CFTR; Chang et al., 2002). PKA and PKC promote the translocation to the apical membrane and activity of the transient receptor potential vanilloid-4 cation channel (TRPV4; Mamenko et al., 2013) and PKA and PKC have an additive effect in regulating the surface expression of the Na, K-ATPase pump (Kristensen et al., 2003). Protein kinases could also indirectly regulate MLC1 export toward the plasma membrane by their involvement in the formation of coat protein II (COPII)-coated vesicles (Farhan et al., 2010). The COPII protein complex forms transport vesicles from the ER and, by binding specific diacidic motifs present in the C terminal of the cargo protein, incorporates it into these vesicles (Barlowe, 2003). The presence of the canonical COPII binding motif DXE in the COOH terminal of the MLC1 protein (**Figure 1**) suggests that the same regulation takes place for MLC1, similarly to what observed for some ion channels and transporters (Wang et al., 2004; Sivaprasadarao et al., 2007; Zhang et al., 2011). The presence of several additional putative phosphorylation sites in the MLC1 sequence, as reported in specific databases (NetPhos 2 Server, Technical University of Denmark; **Figure 1**), and the identification by LC-MS analysis of several kinases and phosphatases among MLC1 interactors in primary rat astrocytes (**Table 1**; Ambrosini manuscript in preparation) suggest a more complex phosphorylation-mediated regulation of MLC1 localization, and probably function, in astrocytes. Experiments are in progress in our laboratory to better understand the role of kinases in MLC1 protein stability, molecular interactions, and function. This knowledge could be relevant for the therapy of MLC since several kinases are currently under investigation as drug targets to modulate the activity of ion channels involved in tumoral, inflammatory, and neurologic diseases (Son et al., 2013).

**Table 1:** **Newly identified megalencephalic leukoencephalopathy with subcortical cyst (MLC1) molecular interactors**.

Kinases and phosphatases	Accession number
Serine/threonine-protein kinase mTOR	P42346
Integrin-linked protein kinase	Q99J82
cAMP-dependent protein kinase catalytic subunit alpha	P27791
Cell division protein kinase 4	P35426
Casein kinase II subunit alpha	P19139
Casein kinase II subunit beta	P67874
Serine/threonine-protein phosphatase PP1-alpha catalytic subunit	P62138
Serine/threonine-protein phosphatase 2A catalytic subunit alpha isoform	P63331
Serine/threonine-protein phosphatase PP1 isozyme 1	P30366
Tyrosine-protein phosphatase non-receptor type 9	Q641Z2
Tyrosine-protein phosphatase non-receptor type 1	P20417
Serine/threonine-protein phosphatase 2A 55 kDa regulatory subunit B alpha isoform	P36876
Tyrosine-protein phosphatase non-receptor type 6	P81718
Serine/threonine-protein phosphatase PP2A-1 catalytic subunit	Q07099
Receptor-type tyrosine-protein phosphatase C	P04157
Receptor-type tyrosine-protein phosphatase alpha	Q03348
Receptor-type tyrosine-protein phosphatase zeta	Q62656
**Ion channel/transporter proteins**	
Voltage-dependent anion-selective channel protein 1	Q9Z2L0
Voltage-dependent anion-selective channel protein 2	P81155
Voltage-dependent anion-selective channel protein 3	Q9R1Z0
Chloride intracellular channel protein 4	Q9Z0W7
Excitatory amino acid transporter 1	P24942

*This table lists some of the newly identified MLC1 interactors among kinase/phosphatase and ion channel/transporter proteins (Ambrosini manuscript in preparation). Interactors were identified by pull-down and LC-MS analysis of primary rat astrocytes. Validation experiments are ongoing.*

### MLC1 Expression in Mouse and Human Brain

One of the few certainties on MLC1 protein is its predominant expression in brain astrocytes, particularly in astrocytic end-feet contacting cerebral blood vessels. Due to this finding, MLC1 is emerging as a promising marker for perivascular astrocytes (Masaki et al., 2012). The first evidence of MLC1 expression in glial cells came from an *in situ* hybridization study in rat brain showing that MLC1 mRNA is mainly expressed in multipotent neural precursor cells during the pre- and peri-natal period, and in astrocytes, Bergmann glia, and ependymal cells, but not oligodendrocytes and neurons, in the adult brain (Schmitt et al., 2003). These researchers also found that MLC1 is developmentally regulated in the mouse brain, being maximally expressed at day 5 after birth and then decreasing to a lower and stable level from post-natal day 7 to adulthood. More recently, the temporal correspondence between the expression of MLC1 in human and mouse brain has been demonstrated (Dubey et al., 2015). Using specific antibodies, MLC1 protein was found predominantly in astrocyte compartments contacting the brain barriers, such as the astrocyte end-feet contacting blood vessels, the meninges (pial membrane) and ependymal cells lining the ventricles, and in Bergmann glia in the cerebellum, but not in neurons, oligodendrocytes, microglia, and endothelial cells (Teijido et al., 2004; Boor et al., 2005, 2007; Ambrosini et al., 2008). Outside the CNS, MLC1 is expressed in blood cells, like lymphocytes, monocytes, and macrophages differentiating *in vitro* (Boor et al., 2006; Petrini et al., 2013). Accordingly, defects in hyposmosis-induced Ca^2+^ influx (see below) were recorded in monocyte-derived macrophages from MLC patients, though the impact of such alterations is unknown since MLC patients do not show overt deficits in blood or immune system functions.

### Intracellular Distribution and Trafficking of MLC1 Protein in Astrocytes

**FIGURE 2:**
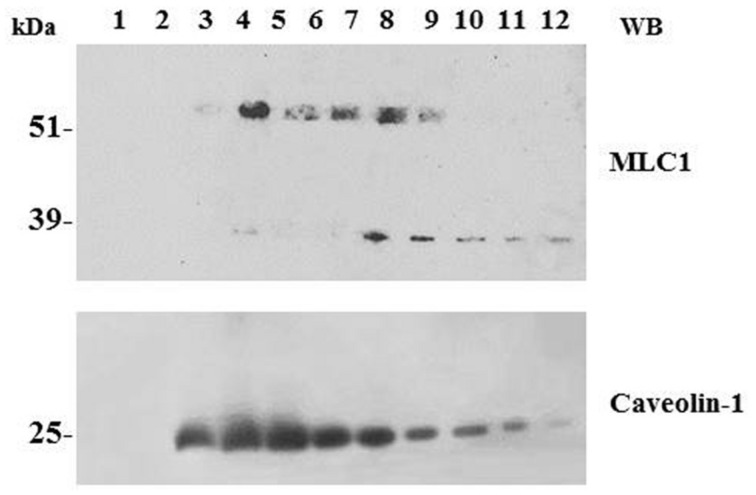
**Association of MLC1 with detergent resistant membrane (DRM)/lipid raft cell compartments**. WB analysis of cholesterol-rich (triton insoluble fractions: 3–6) and cholesterol-poor membrane fractions (triton soluble fractions: 10–12) of WT MLC1 expressing U251 cells. The 60–64 kDa MLC1 component is associated with the DRM fractions, whereas the 30 kDa band is detected only in the soluble fractions together with a portion of the 60–64 kDa component. The same membrane was blotted with anti-caveolin-1 antibody to track the position of caveolae-enriched membranes in the DRM fractions.

Biochemical studies in cultured astrocytes and rat brain tissue showed that MLC1 is present in specialized areas of the plasma membrane called lipid rafts (Lanciotti et al., 2010). Lipid rafts are microdomains of the plasma membrane formed by a tightly ordered lipid phase that is enriched in sterols (including cholesterol), sphingolipids (including gangliosides) and phospholipids with saturated hydrocarbon chains (Wang and Paller, 2006). Lipid rafts are defined by resistance to extraction with non-ionic detergents and low density in sucrose gradients. These microdomains are highly dynamic and possess considerable lateral mobility within the loosely ordered membrane. Caveolar rafts containing caveolae, stable flask-shaped invaginations of the plasma membrane, are a subtype of lipid rafts highly enriched in cholesterol and coated by caveolin-1 (cav-1). Cav-1, a hairpin-like palmitoylated structural protein, is thought to stabilize the invaginated caveolar structure. Caveolae have been described in several cell types, including astrocytes and astrocytoma/glioma cells (Zschocke et al., 2005; González et al., 2007), and are involved in protein trafficking and formation of cell signaling complexes (Simons and Ikonen, 1997; Brown, 2006). By performing lipid raft separation by sucrose gradient, we found that only the MLC1 dimers are distributed in the membrane caveolar raft fraction, both in cultured astrocytes and in rat brain tissue (Ambrosini et al., 2008; Lanciotti et al., 2010; **Figure 2**). Presence of MLC1 protein in caveolar rafts is supported by binding of cav-1 to the NH_2_ intracellular terminal of the MLC1 protein containing the putative caveolin/cholesterol binding site (Lanciotti et al., 2010; **Figure 1**).

**FIGURE 3:**
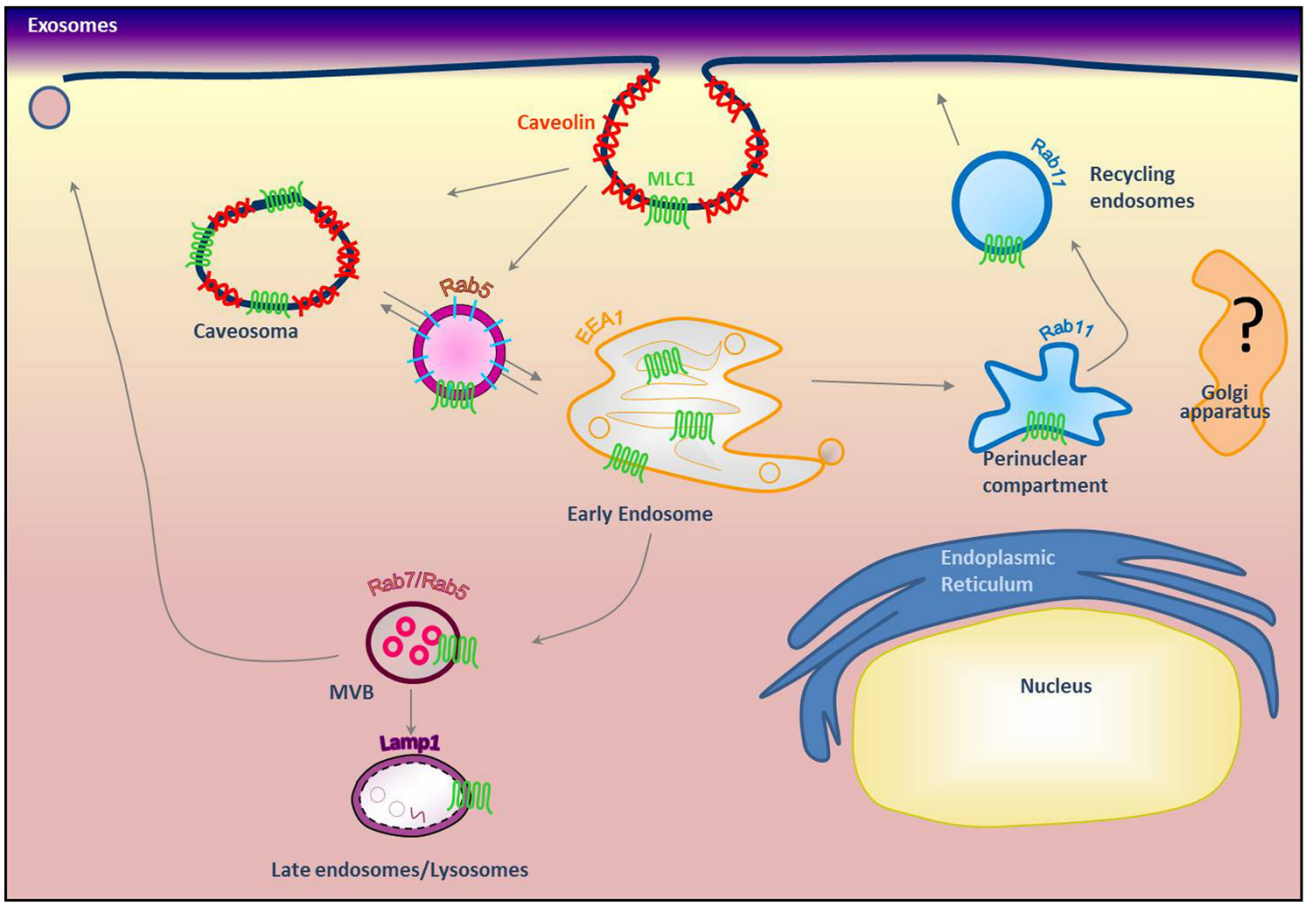
**Modeling of MLC1 intracellular trafficking**. Our previous results indicated that MLC1 is internalized via caveolae-mediated endocytosis and traffics through Rab5^+^ and EEA1^+^ early endosomes where it is sorted to the recycling (Rab11^+^) or degradative pathway (Lamp-1^+^; Lanciotti et al., 2010; Brignone et al., 2014) (modified from Brignone et al., 2014).

**FIGURE 4:**
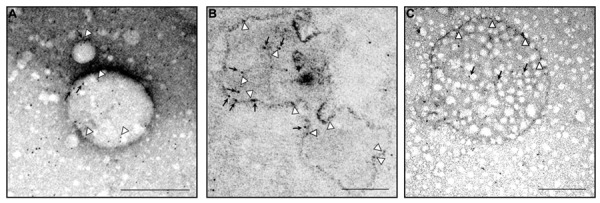
**Ultrastructural analysis of endogenous MLC1 localization in early endosome compartment (EEA1^+^ and Rab5^+^ organelles) and in a multivesicular body**. Electron microscopy analysis of ultracentrifuged cytosolic protein fractions obtained from cultured rat astrocytes was performed using anti-MLC1 Ab (10 nm gold particles) in combination with anti-EEA1 or anti-Rab5 Abs (5 nm gold particles). **(A)** MLC1 (black arrows) is localized in Rab5^+^ organelles (arrowheads) **(B)** MLC1 is localized in the membrane and intraluminal areas (black arrows) of EEA1^+^ organelles (arrowheads). **(C)** MLC1 immunostaining in the membrane (arrowheads) and intracellular vesicles (black arrows) of a multivesicular body-like structure. Scale Bars: 200 μm.

Because MLC1 function is still unknown, we do not understand at present the functional consequences of MLC1 localization in caveolar rafts. However, we have found that cholesterol and agents modulating caveolae-dependent endocytosis influence MLC1 transport from the plasma membrane to the intracellular compartments (Lanciotti et al., 2010). Our studies demonstrated that in astrocytes exposed to hyposmotic stress MLC1 is translocated to the plasma membrane and then internalized by caveolar endocytosis to be sorted to recycling or degradation pathways (**Figure 3**). By cell fractionation, immunofluorescence and electron microscopy analysis of rat primary astrocytes and human astrocytoma cells, MLC1 protein was detected in early endosomes (EEA1^+^ and Rab5^+^; **Figures 3** and **4A,B**; Lanciotti et al., 2010; Brignone et al., 2014) and in Lamp-1^+^ organelles and multivesicular bodies (**Figure 4C**; Lanciotti et al., 2010; Brignone et al., 2014), but not, or at very low levels in Golgi apparatus and clathrin vesicles (Lanciotti et al., 2010). More recently, we found that in unstimulated astrocytes MLC1 is abundantly expressed in the Rab11^+^ perinuclear storage/recycling compartment from which it is recruited back to the plasma membrane when cells are exposed to hyposmotic stress (**Figure 3**; Brignone et al., 2014). Cytoskeletal elements are involved in MLC1 intracellular trafficking since nocodazole treatment, which perturbs microtubule organization, hampers MLC1 accumulation in the Rab11^+^ endosomal recycling compartment in rat primary astrocytes (Lanciotti et al., 2010). It has been shown that stabilization of MLC1 in the plasma membrane of rat astrocytes cultured for 3 weeks in the presence of the antimitotic cytosine arabinoside, which leads to astrocyte maturation/differentiation, depends on intact actin filaments, but not microtubules or intermediate filaments (Duarri et al., 2011). In this context, post-translational modifications can exert an important role in MLC1 forward trafficking and stabilization in the plasma membrane (see above). Overall, these results provide evidence that the membrane expression level of MLC1 is spatially and temporally regulated by agents modulating intracellular trafficking, cytoskeletal organization and lipid composition of the plasma membrane, as observed for many ion channels and transporters (Dart, 2010; Curran and Mohler, 2014).

## MLC1 Molecular Interactors

One of the first steps to disclose the function of a novel protein is the identification of its molecular interactors, which can provide information on the biochemical pathways in which the protein of interest is involved (**Table 2**). By using this approach, *GlialCAM* has been identified as the second MLC disease-associated gene and the molecular chaperon that targets MLC1 to astrocyte cell–cell junctions (López-Hernández et al., 2011a,b; Capdevila-Nortes et al., 2013).

**Table 2:** **List of described MLC1 molecular interactors in astrocytes**.

	Experimental techniques	Interaction compartment	Experimental models	Reference
GlialCAM	Quantitative proteomic analysis and quantitative mass spectrometry; *in situ* hybridization and immunohistochemistry (IHC); immunofluorescence (IF); electron microscopy (EM) immunostaining	Membrane (COOH)	Primary rat astrocytes; HeLa cells Mlc1 (-/-) zebrafish Mlc1 (-/-) mice Human brain tissue	López-Hernández et al. (2011a,b), Capdevila-Nortes et al. (2013), Arnedo et al. (2014a,b), Barrallo-Gimeno and Estévez (2014), Sirisi et al. (2014), Dubey et al. (2015)
Na,K-ATPase beta1	Yeast two-hybrid assay; Ouabain-affinity chromatography; Co-purification of His-tagged proteins and LC–MS analysis; IF; IHC	Membrane/cytosol (NH_2_)	Rat astrocytes and rat brain; human astrocytoma cells	Brignone et al. (2011), Brignone et al. (2014), Lanciotti et al. (2012)
TRPV4	Pull-down assay; ouabain-affinity chromatography.	Membrane	Rat astrocytes; human astrocytoma cells	Lanciotti et al. (2012), Brignone et al. (2014)
V-ATPase	Co-purification of His-tagged proteins and LC-MS analysis; IF; IHC	Cytosol	Rat primary astrocytes; human astrocytoma cells; human brain tissue	Brignone et al. (2014)
DGC complex	IF; IHC; immunoprecipitation (IP); Co-purification of His-tagged proteins and co-fractionation assays; ouabain-affinity chromatography	Membrane/cytosol	Gliotic brain tissue; glioblastoma tissue and brain tissue from an MLC patient; human and rat astrocytes; rat brain extracts; human astrocytoma cells	Boor et al. (2007), Lanciotti et al. (2010, 2012), Brignone et al. (2011)
ZO-1	EM immunostaining; IP	Membrane	Rat and human brain	Duarri et al. (2011)
Kir4.1	IP; co-purification of His-tagged proteins and co-fractionation assays; ouabain-affinity chromatography; IF; IHC	Membrane	Human and rat astrocytes; rat brain extracts; human astrocytoma cells	Boor et al. (2007), Lanciotti et al. (2010, 2012), Brignone et al. (2011), Dubey et al. (2015)
Cav-1	Pull-down assay; co-purification of his-tagged proteins; IF	Membrane (NH_2_; COOH)	Rat astrocytes; human astrocytoma cells	Lanciotti et al. (2010)
ClC2	IHC; EM		Mlc1-null mice	Hoegg-Beiler et al. (2014), Dubey et al. (2015)

Recently, new findings obtained in knock-out (KO) animal models have allowed to better understand the relationship between GlialCAM and MLC1 *in vivo* (see below). As this article focuses on the role of MLC1 protein, we refer the reader to specific publications for detailed studies on GlialCAM involvement in MLC disease (López-Hernández et al., 2011a,b; Capdevila-Nortes et al., 2013).

### Dystrophin–Dystroglycan Complex and Junctional Proteins

**FIGURE 5:**
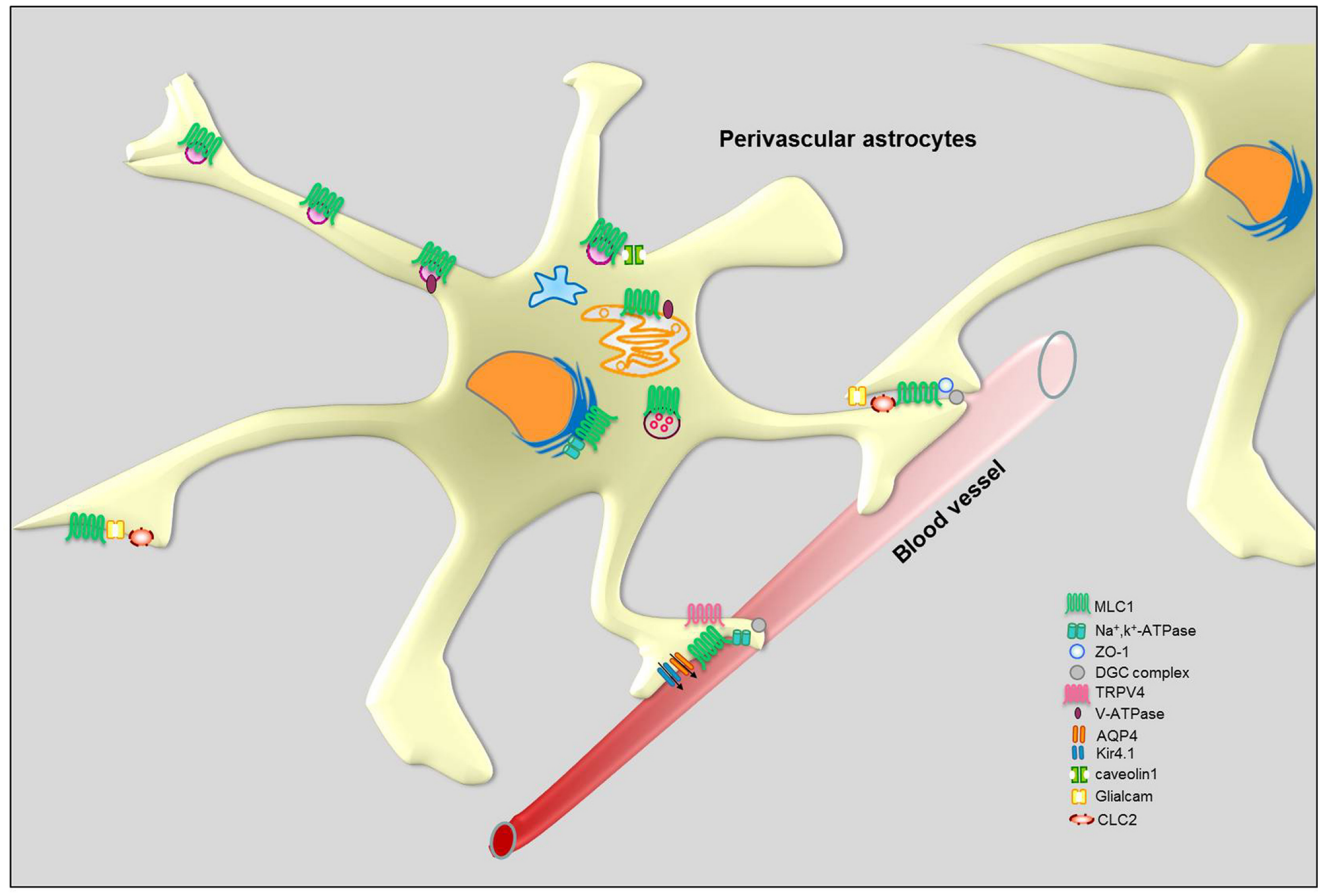
**Schematic representation of MLC1 molecular interactions in perivascular astrocytes**.

The observation that MLC disease shares some pathological features with congenital muscular dystrophies with brain involvement caused by mutations in proteins of the dystroglycan (DG) associated protein complex dystrophin–dystroglycan complex (DGC; Boor et al., 2007) prompted us and other research groups to investigate the relationship between MLC1 and DGC. The DGC is a multiprotein complex highly expressed in astrocyte end-feet where it stabilizes ion and water channels, like the inward rectifier potassium 4.1 (Kir4.1) channel and the water channel aquaporin-4 (AQP4; Noell et al., 2011). It has been shown that in the human brain MLC1 co-localizes with DGC proteins, like α/β-DG, syntrophin and agrin, in astrocyte end-feet contacting blood vessels (Boor et al., 2007; Ambrosini et al., 2008). Moreover, in brain tissue from a patient with MLC some components of the DGC (Kir4.1, agrin, and α-DG) are mislocalized while others (merosin, β-DG and AQP4) retain their normal perivascular localization (Boor et al., 2007). These results appeared of interest since agrin and α-DG are specifically involved in Kir4.1 clustering in the glial cell plasma membrane (Noël et al., 2005) and deletion of the *Kir4.1* gene in mice causes myelin vacuolation and cyst formation that are reminiscent of the MLC phenotype (Neusch et al., 2001). The MLC1/DGC protein interaction was also supported by biochemical experiments showing that Kir4.1 and MLC1 co-immunoprecipitate in human brain (Boor et al., 2007) and that MLC1 is detected among DGC complex proteins in cultured rat and human astrocytes (Boor et al., 2007; Ambrosini et al., 2008; Lanciotti et al., 2010). In contrast with these data, electron microscopy showed that MLC1 co-localizes with the zonula occludens protein-1 (ZO-1), a protein present in astrocyte–astrocyte junctions, but not with β-DG in astrocyte end-feet contacting cerebrovascular endothelial cells in the normal human adult cerebellum (Duarri et al., 2011; **Figure 5**). The same study reports that MLC1 does not co-immunoprecipitate with DGC components in cultured rat astrocytes acquiring a differentiated morphology upon prolonged treatment with cytosine arabinoside (Duarri et al., 2011). Differences in MLC1 molecular interactions in different brain regions (cerebral cortex versus cerebellum) and/or astrocyte developmental stages (proliferating neonatal rat astrocytes versus mature differentiated astrocytes) could account for these discrepancies. Interestingly, the DGC itself is also involved in the organization and maintenance of junctional complexes during brain development (Nico et al., 2003, 2004; Sjö et al., 2005). Further studies are needed to clarify the issue of MLC1/DGC relationship.

### MLC1 Molecular Interactors Involved in Osmoregulation and pH Control

Consistent with the hypothesis that MLC1 plays a role in astrocyte-mediated osmoregulation, the MLC1 molecular interactors we identified in preliminary studies included ion channels and accessory proteins that are enriched in astrocyte end-feet (**Table 2**). Using complementary biochemical, proteomic and *in vivo* protein interaction assays (like pull-down, yeast two-hybrid system assays, His co-fractionation associated to LC-MS analysis and ouabain affinity chromatography) in cultured rat astrocytes and human astrocytoma cells, we found that MLC1 binds the β1 subunit of the Na, K-ATPase enzyme (Brignone et al., 2011). We also showed that MLC1 is part of a multiprotein complex comprising Kir4.1, cav-1, TRPV4, AQP4, syntrophin, and the vacuolar ATPase (V-ATPase) and is involved in the regulation of ion transport, cellular volume changes and intraorganelle pH control (Brignone et al., 2011, 2014; Lanciotti et al., 2012). We have found that other ion channels/transporters interact with MLC1, such as the chloride intracellular channel 4 (CLIC4), the voltage-dependent mitochondrial anion channels (VDAC1-3) and the excitatory amino acid transporter 1 (EAAT1; **Table 1**). The functional relationship of these molecules with MLC1 is under investigation. Cell fractionation and immunolocalization experiments revealed that in astrocytes MLC1 interaction with Na, K-ATPase and V-ATPase occurs mainly in the membrane of intracellular organelles, like early and recycling endosomes, in basal culture conditions and also in the plasma membrane after exposure to osmotic stress (Brignone et al., 2011, 2014). These results indicate that macromolecular complexes comprising MLC1 may modulate the astrocyte response to osmotic imbalance by spatially operating in the plasma membrane and in intracellular membranes, similarly to what observed for protein complexes linked to Ca^2+^, K^+^, and Cl^-^ channels (Babcock and Li, 2013; Venkatachalam et al., 2014). A more systematic evaluation of the function and mutation-induced alterations of MLC1 in different membrane compartments should be pursued also in view of possible therapeutic applications. In fact, there is increasing evidence that pharmacological treatments can differently target ion channel and transporter protein complexes in the plasma membrane or intracellular compartments (Babcock and Li, 2013).

## Deciphering MLC Pathogenesis: From Cellular to Animal Models

To shed light on MLC1 function and its role in MLC pathogenesis, in the last years different *in vitro* and *in vivo* pathological models have been developed. *In vitro* MLC disease models were set up by overexpressing WT and mutated MLC1 in heterologous cells and astrocytes or by knocking-down MLC1 in astrocytes. Earlier studies performed in heterologous cells (HeLa cells, HEK293 cells, Xenopus oocytes) expressing mutated MLC1 protein showed that many missense pathological mutations impaired MLC1 translocation to the plasma membrane. MLC1 mutations also affected the protein half-life by causing an increase in protein degradation (Teijido et al., 2004; Duarri et al., 2008). This effect was mainly ascribed to protein misfolding problems and activation of the endoplasmic reticulum associated degradation pathway (ERAD) since ERAD inhibitors could block MLC1 degradation. After the discovery that cultured astrocytes express MLC1 protein (Ambrosini et al., 2008), these cells became the most relevant *in vitro* model to study MLC1 pathophysiology. Interestingly, some MLC1 mutations behave differently when overexpressed in rat astrocytes compared to heterologous cells (Duarri et al., 2008), highlighting the need to investigate MLC1 mutation-induced phenotypes in an astrocyte background. To this end, we selected human astrocytoma cells (U251 cells), since these cells express almost undetectable levels of endogenous MLC1 protein. We therefore generated U251 cell lines stably expressing MLC1 WT or carrying some of the several missense mutations found in MLC patients (Brignone et al., 2011). The obtained astrocytoma cell lines expressed MLC1 at levels comparable to those detected in primary rat astrocytes.

Using U251 cell lines expressing different MLC1 pathological mutations (i.e., S426R, S280L, and C125R) we found that not all the mutated proteins were retained in the ER. For example, MLC1 protein carrying the S426R mutation was able to reach the plasma membrane, similarly to WT MLC1 (Brignone et al., 2011). This result suggested that *in vitro* modeling of MLC1 pathological mutations could unravel MLC1 mutation-specific functional defects and eventually prove useful for testing patient-tailored therapies. Using this system, we also found that the analyzed missense mutations altered the interaction of MLC1 with some previously identified interactors, such as GlialCAM, Kir4.1, Na, K-ATPase, V-ATPase, and TRPV4 channel (Brignone et al., 2011; Lanciotti et al., 2012), supporting further the functional involvement of MLC1 in this macromolecular complex. Interestingly, downregulation of MLC1 in primary rat astrocytes by small interfering RNA (siRNA) silencing induced astrocyte vacuolation, a pathological feature found in the MLC-affected brain (Duarri et al., 2011) and in *mlc1*-null mice (Dubey et al., 2015), but did not alter the localization of the MLC1 interactor ZO-1 (Duarri et al., 2011). Collectively, these results indicate that *in vitro* modeling of MLC disease can, at least in part, recapitulate MLC1 dysfunction.

Two different mouse models have been recently developed in which MLC1 has been constitutively knocked-down in all tissues (Hoegg-Beiler et al., 2014; Dubey et al., 2015). A zebrafish model has also been generated by deleting the *MLC1* ortholog gene (Sirisi et al., 2014). MLC1-null mice recapitulate some features of the human MLC disease, such as early-onset megalencephaly and augmented brain water content, with appearance of aberrant astrocytic processes adjacent to fluid/brain barriers and progressive white matter vacuolization. However, both KO mice do not develop motor disabilities (even at later stages of life) or brain cysts that are characteristic of the human disease. In general, when neuropathological hallmarks were compared, brain lesions in MLC1-null mice were less severe than those in MLC-affected brain and zebrafish lesions were even less pronounced than mice lesions, predicting interspecies differences, or occurrence of compensatory mechanisms in KO animals. Analysis of *mlc1*-null mice revealed that, contrary to what was found *in vitro* (López-Hernández et al., 2011b), MLC1 is important for the correct localization of GlialCAM and ClC-2 in astrocytes. It has been shown that MLC1 also acts *in trans,* by influencing GlialCAM expression in oligodendrocytes which are devoid of MLC1 (Hoegg-Beiler et al., 2014). On the contrary, lack of MLC1 does not affect the distribution of α/β-DG, agrin, Na, K-ATPase, TRPV4, and ZO-1 in astrocyte perivascular end-feet (Dubey et al., 2015). Evidence supporting the importance of MLC1 for the correct localization of GlialCAM and ClC-2 was also obtained in cultured astrocytes derived from *mlc1*-null mice that were exposed to a high extracellular K^+^ concentration mimicking neuronal activity (Hoegg-Beiler et al., 2014; Sirisi et al., 2014). However, immunohistochemical analysis of brain sections from a MLC patient carrying a missense mutation (S69L) leading to MLC1 downregulation showed no apparent abnormalities in GlialCAM localization in perivascular astrocytes (López-Hernández et al., 2011b); in the same patient GlialCAM mislocalization was found restricted to the Bergman glia in the cerebellum (Sirisi et al., 2014).

Although KO animal models do not completely recapitulate the human disease, they confirm that astrocyte-mediated defects in water fluxes and cell volume regulation are involved in MLC disease pathogenesis, as suggested by results in *in vitro* models. Overall these findings indicate that the experimental models used to study MLC1 function and MLC disease pathogenesis can provide different (sometimes contradictory) results. Some discrepancies may reflect the inability of the *in vitro* systems to reproduce the complexity of astrocyte functional and structural connections *in vivo.* It is generally accepted that gene inactivation *in vivo* represents an essential approach to study the physiological roles of several proteins. However, KO mice, while useful for a rough characterization of gene function, have some disadvantages for deciphering precisely pathogenetic mechanisms, including gene redundancy, and developmental issues. Furthermore, not all the MLC1 mutations analyzed *in vitro* lead to complete protein degradation, a phenotype recapitulated by null-mouse models. Noteworthy, astrocytes display heterogeneous characteristics depending on species of origin, brain region, developmental stage, environmental factors, and disease states, all of which may render experimental results highly variable. The complexity of the glia themselves as well as the marked increase in the ratio of glia to neurons in higher mammals (Pfrieger, 2009) suggest that some roles of glia studied in mouse models might be non conserved or even more critical in primates and humans (Molofsky et al., 2012). For these reasons, advantages and disadvantages of each experimental system should be taken into account for a correct evaluation of the results obtained.

## Astrocyte Functional Pathways Affected by MLC1 Mutations

### Regulation of Ca^2+^ Influx

Intracellular Ca^2+^ changes participate in the regulation of regulatory volume decrease (RVD) that occurs after cell swelling in astrocytes. It has been shown that the cation channel TRPV4 co-localizes with AQP4 in the astrocyte end-feet and is responsible for an outward-rectifying Ca^2+^ conductance and Ca^2+^ influx following cell swelling induced by mechanical and osmotic stress (Benfenati et al., 2007, 2011; **Figure 5**). The TRPV4-mediated Ca^2+^ influx is an essential step in the activation of RVD in astrocytes which is needed to rescue the temporary cell swelling induced by hyposmosis. Using the U251 astrocytoma cell model, we found that MLC1 mutations causing retention of the protein in the cytoplasmic perinuclear area affect TRPV4-mediated Ca^2+^ influx induced by hyposmotic stress (Lanciotti et al., 2012). This finding supports the hypothesis that MLC1 localized in the plasma membrane can functionally cooperate with TRPV4 to regulate Ca^2+^ influx and RVD after osmotic imbalance. Moreover, as in primary astrocytes (Benfenati et al., 2007, 2011), also in astrocytoma cells hyposmotic stress induces heterogeneous Ca^2+^ transients that differ for lag time of onset, profile of the transient (i.e., peak shaped, sustained, or a combination of the two) and maximal amplitude of the Ca^2+^ changes (Lanciotti et al., 2012). The high variability in the Ca^2+^ response is hardly compatible with a direct effect of swelling on TRPV4 channels, but more likely is the result of an indirect effect mediated by a different molecular sensor (Christensen and Corey, 2007). The TRPV4 cation channel is known as a polymodal transducer of mechanical stretch, osmotic pressure, and warmth perception (Nilius and Voets, 2013). However, it is debated whether TRPV4 is itself sensitive to such stimuli or lies downstream of an osmotic sensor and only mediates the transduction of the stimulus. The available evidence indicates that TRPV4 can be activated by second messengers and phosphorylations (Xu et al., 2003; Poole et al., 2013). It has been shown that activation of TRPV4 depends on phosphorylation on tyrosine 253 by Src kinase or on 5,6′-epoxyeicosatrienoic acid (5,6′-EET) resulting from the breakdown of arachidonic acid by cytochrome P450 epoxygenase (Xu et al., 2003; Poole et al., 2013). Notably, our recent unpublished data indicate that MLC1 can bind to a number of proteins with enzymatic activity, like kinases and phosphatases (**Table 1**). Further studies are needed to understand if MLC1 affects TRPV4 function by acting as a docking site for mechanical sensors. We recently demonstrated that MLC1, by modulating endosome acidity (see below), can influence TRPV4 trafficking favoring channel recycling versus degradation. We cannot exclude that this effect might mechanistically explain the functional cooperation between MLC1 and TRPV4. Accordingly, defects in calcium influx during hyposmosis were also observed in monocyte-derived macrophages from MLC patients (Petrini et al., 2013).

### Regulation of Cl^-^ Channel Function

Among the processes involved in cell volume regulation chloride currents are the object of intense research aimed at understanding their involvement in the pathophysiology of MLC1. Chloride is largely used as co-ion together with cations for electrical neutralization during membrane ion fluxes or as a counter-ion in anion-exchange mechanisms. According to Walz (2002) the cytoplasmic chloride concentration in cultured astrocytes is around 40 mM. *In vivo* studies generated conflicting results. Depending on the brain area, the intracellular chloride concentration ranged between 36 and 46 mM (Smith et al., 1981), while a lower concentration (6 mM) was found in unspecified glial cells in slice preparations (Ballanyi et al., 1987). It seems that oligodendrocytes have a lower intracellular chloride concentration compared to astrocytes, in which the chloride intracellular concentration is actively kept around 40 mM. In astrocytes the resulting reversal potential, which is more positive than the resting potential, gives rise to an eﬄux of the ion upon opening of chloride channels during volume regulatory events (Walz, 2002).

Based on the available evidence, two different chloride currents are potentially involved in MLC pathology. Defects in a RVD-induced chloride current have been noted in rat astrocytes following siRNA-mediated MLC1 downregulation and in MLC patient-derived lymphoblasts (Ridder et al., 2011). Based on activation by hypotonic shock, ion selectivity, pharmacological sensitivity, and outward rectifying property, the MLC1-dependent current was identified as a volume regulated anion current (VRAC). VRAC currents were also studied in astrocytes derived from *mlc1*-null mice (Sirisi et al., 2014; Dubey et al., 2015). In one of these studies, a VRAC current was identified as the Cl^-^ current sensitive to the VRAC inhibitor DCPIB (Sirisi et al., 2014). The hypotonicity-induced VRAC current amplitude in MLC1^-/-^ astrocytes differed slightly, but not significantly, from that recorded in WT astrocytes. However, the slightly smaller VRAC current in MLC1^-/-^ astrocytes was associated with more pronounced vacuolation in brain tissue, consistent with a functional decay of water/ion regulatory mechanisms (Sirisi et al., 2014). In another study performed in astrocytes derived from *mlc1*-null mice where the *MLC1* gene has been replaced by the green fluorescent protein encoding sequence, astrocytes exhibited a hypotonic challenge-induced Cl^-^ current that was identified as due to VRAC channels by cadmium insensitivity and tamoxifen sensitivity (Dubey et al., 2015). Such a current had a lower amplitude in astrocytes from MLC1^-/-^ mice compared to those from wild-type mice, indicating a VRAC current functional decay due to MLC1 ablation. Consistent with the role of VRAC in RVD, astrocytes from MLC1^-/-^ mice reacted to a hypotonic challenge with a slower RVD (Dubey et al., 2015). It is not clear whether differences in the detection of the VRAC-induced chloride current in the two *mlc1*-null mice models are due to technical problems or to the influence of the genetic background (C57BL/6 versus C57BL/6-129/Svj mixed genetic background). VRAC is not only involved in the RVD, but also in pathological events like apoptotic volume decrease, necrotic volume increase following lactacidosis and excitotoxicity (Okada et al., 2009; Pedersen et al., 2015). Notably, VRAC is the target of several regulatory mechanisms activated by osmotic cell swelling; among these, are EGFR activation and phosphorylation by PI3K, src and ERK (Pedersen et al., 2015). Data supporting the possibility that MLC1 protein itself acts as VRAC are not available. Recently, LRRC8A, a member of the four-transmembrane protein family, was recognized as a component of the VRAC channel in non-neural cells (Qiu et al., 2014; Voss et al., 2014) and also in glial cells were LRRC8A is an indispensable component of a permeability pathway that mediates both swelling-activated and agonist-induced amino acid release (Hyzinski-García et al., 2014). However, the relationship between MLC1 and LRRC8A is still unknown.

Based on the presence of white matter vacuolation in mice lacking ClC-2 (Blanz et al., 2007), this chloride channel was considered as potentially involved in MLC1 disease but no ClC-2 mutations were found in MLC patients (Scheper et al., 2010). However, although initially in humans ClC-2 loss- and gain-of function mutations were linked to idiopathic generalized epilepsy with no apparent leukodystrophy (Haug et al., 2003), a recent study has revealed that autosomal-recessive *CLCN2* mutations cause a leukoencephalopathy characterized by intramyelinic oedema (Depienne et al., 2013).

ClC-2 channels are almost ubiquitous and their whole-cell current is characterized by hyperpolarization-induced slow activation, slow deactivating tail currents at more positive potentials, strong inward rectification, and blockade by iodide but not by classical Cl^-^ channel inhibitors and tamoxifen (Jentsch et al., 2002). In the first *in vitro* study aimed at elucidating the role of ClC-2 channels in MLC, no correlations between MLC1 protein and ClC-2 were found, MLC1 mRNA interference did not change ClC-2 level, and the two proteins did not co-precipitate (Jeworutzki et al., 2012). In the same study, attention was also focused on GlialCAM, whose mutations were found in a subpopulation of MLC1 patients (López-Hernández et al., 2011a,b; Capdevila-Nortes et al., 2013). Overexpression of GlialCAM together with ClC-2 in Xenopus oocytes caused remarkable changes of current features, such as ohmic conductance instead of inward-rectification, instantaneous activation instead of slow activation, and large increase in the current amplitude (Jeworutzki et al., 2012). However, the current recorded in primary astrocytes and identified as carried by ClC-2 because of lack of permeability to iodide and insensitivity to tamoxifen, did not show the above features even if cultured astrocytes are known to express GlialCAM. It cannot be ruled out that the abrupt changes recorded in the heterologous system are the result of GlialCAM protein overexpression (Jeworutzki et al., 2012). A recent study performed in parallel in three different KO mouse models, MLC1^-/-^, GlialCAM^-/-^ and ClC-2^-/-^, addressed this point with a detailed analysis of whole-cell recordings from slice preparations (Hoegg-Beiler et al., 2014). As in primary cortical astrocytes obtained from the previously characterized mouse models (Sirisi et al., 2014), also in WT Bergman glia, known to express GlialCAM, Cl^-^ currents showed features expected in the absence of GlialCAM (i.e., inward rectification and slow activation). Moreover, ablation of GlialCAM did not result in the expected changes (i.e., large decrease of the amplitude and stronger inward rectification) and caused a mild decrease of the current, compatible with the decrease in ClC-2 expression observed in MLC1^-/-^ and GlialCAM^-/-^ mice by immunohistochemistry. So far, the most likely interpretation of these results is that MLC1 plays a role as a chaperone contributing to the correct localization of ClC-2 proteins, but is not involved in the modulation of the biophysical features of ClC-2 channel.

### pH Regulation in the Endosomal Compartment

Both the V-ATPase and the Na, K-ATPase pumps are known to regulate acidity of the endosomal compartment. Aiming at investigating further the functional relationship between MLC1 and V-ATPase/Na, K-ATPase in the context of the MLC1-associated macromolecular complex described in *in vitro* experiments (Brignone et al., 2011, 2014), we studied the effects of MLC1 mutations on organelle pH.

Using a video-imaging approach with the pH sensitive dye FITC-dextran, we found that in astrocytoma cells expressing WT, but not mutated MLC1, early endosomes (i.e., EEA1^+^ and RAB5^+^) showed a higher pH compared to early endosomes from the parental U251 cells (Brignone et al., 2014). This result is compatible with a possible role for MLC1 in the modulation of V-ATPase and Na, K-ATPase function. Being the early endosomal compartment a check-point station where proteins are sorted toward recycling or degradation depending on further acidification of the organelles, these results suggest that MLC1 can influence the trafficking of proteins co-localizing in the same early endosomes. Indeed, we found that WT but not mutated MLC1 favored TRPV4 channel recycling (Brignone et al., 2014). These results are consistent with a functional role of MLC1 in endosome pH regulation, since recycling vesicles are characterized by a slightly less acidic pH compared to vesicles sorted toward the lysosomal degradative pathway (Grant and Donaldson, 2009; Scott and Gruenberg, 2011). From a pathogenic point of view, it is interesting to note that alterations in organelle pH regulation induce endosome enlargement and formation of intracellular vacuoles similar to those observed in MLC1 KO astrocytes (Duarri et al., 2011) and that some neurodegenerative diseases are characterized by abnormal giant endosomes (Gouras, 2013). It has been hypothesized that disturbance of the endoplasmic compartment pH might be associated with the development of autistic behavior resulting from an irregular arrangement of membrane components like channels and transporters in the plasmalemma of neurons and astrocytes during development (Kondapalli et al., 2014). Notably, autism is part of the symptomatology of MLC disease (López-Hernández et al., 2011a).

## MLC1 Functions: Astrocyte Specific Ion Channel or Ion Channel Regulator?

The almost exclusive expression of MLC1 in astrocytes together with the neuropathological alterations observed in MLC patients and the results obtained in cellular models have led to hypothesize a role for MLC1 in the regulation of ion/water fluxes controlling cell volume in specific astrocytic districts such as those facing the brain barriers (blood–brain and cerebrospinal fluid–brain barriers).

Does MLC1 directly function as an ion/water permeation site or as a transport protein? Ion channels herein referred to are crucial for the transepithelial transport of salt and water, the regulation of cellular volume and pH, the acidification of intracellular organelles, and chemical signaling. They may be regulated by calcium, pH, phosphorylation, and lipids. Many channels are oligomers of identical or homologous pore-forming α subunits and assemble in complexes with β/γ subunits which may be essential for their function or modulate their properties. Most of these features have been described for MLC1 (see above). Experimental studies revealed that several different (also redundant) mechanisms control MLC1 membrane localization (protein–protein, protein–lipid interactions, post-translational modifications) suggesting that a strict temporal and spatial regulation of MLC1 is necessary in astrocytes. Although at present conclusive results ruling out MLC1 direct involvement in ion/water permeation/transport in astrocytes are missing, the ensemble of the data collected so far converge to delineate a dual role for MLC1 ranging from a chaperoning to a regulatory role of functional events.

The observation that several of the pathological MLC1 mutations cause a mislocalization of the protein together with that of some of its functional interactors suggests a chaperoning-like role of MLC1 in the correct assembly of those specific proteins. On the other hand, the variety of events affected by MLC1 ablation or mutations, together with the recognition of several putative binding sites that could act as docking sites for regulatory enzymes, prompt to consider its regulatory role in the complex scenario of events taking place in specific intracellular domains and involving water/ion movements and pH control.

Besides the exact definition of the multifaceted role played by MLC1, the advent of MLC1 in animals developing myelin prompts to consider in a phylogenetic perspective the importance of this protein for the new needs of such a complex functional structure comprising astrocytes, oligodendrocytes, and neurons. In MLC disease myelin degeneration is not attributable to directmyelin or oligodendrocyte protein alterations but to an indirect effect mediated by astrocytic dysfunction. Although at present the molecular mechanisms causing myelin degeneration in MLC disease are not known it is established that defects in astrocyte maturation, astrocyte functional impairment causing accumulation of toxic substrates, and/or failure of specific astrocyte-mediated homeostatic pathways can affect myelin formation and maintenance leading to cystic or vacuolating myelin degeneration (reviewed in Lanciotti et al., 2013 and references therein). Due to the important role played by astrocytes in regulating oligodendrocyte progenitor cell proliferation, migration, and differentiation during CNS development (Clemente et al., 2013), we cannot exclude that dysfunctional astrocytes could affect myelination also by acting on oligodendrocyte development.

## Concluding Remarks

Channelopathies are a diverse set of disorders associated with defects in ion channel (and transporter) function that are mostly linked with inherited mutations impairing the biophysical properties of the channels. The rapid progress made in molecular genetics and electrophysiology has allowed to expand the field of channelopathies revealing that many disorders reflect dysfunctions in regulatory proteins that alter ion channel synthesis, membrane trafficking and/or posttranslational modifications.

Moreover, associated proteins can define tissue/organ functional specificity, making ion channels at a specific location more susceptible to a pathological condition even if their expression is not tissue-specific. This could be the case of MLC1 which is highly and exclusively expressed by astrocytes in the CNS. The results obtained so far demonstrate MLC1 involvement in astrocyte volume control. Astrocytes not only undergo dramatic volume changes as a result of the ionic dysregulation that occurs in pathological conditions such as ischemia, trauma, and epilepsy but also during neuronal activity in conjunction with extracellular K^+^ buffering (Florence et al., 2012 and references therein). These volume regulatory mechanisms may thus be fundamental for the maintenance of the homeostatic conditions allowing optimal CNS function. A deeper understanding of the structure and function of MLC1 protein and its relationship with ion channels and ion channel-related proteins may contribute not only to identify novel therapeutic approaches for the rare MLC genetic disease, but may also provide insights into the mechanisms underlying more common brain disorders such as migraines, epilepsies, ischemia, and autism.

## Conflict of Interest Statement

The authors declare that the research was conducted in the absence of any commercial or financial relationships that could be construed as a potential conflict of interest.
